# Analysis of belimumab prescription and outcomes in a 10-year monocentric cohort: is there an advantage with early use?

**DOI:** 10.1136/rmdopen-2023-003981

**Published:** 2024-04-12

**Authors:** Chiara Tani, Dina Zucchi, Chiara Cardelli, Elena Elefante, Viola Signorini, Davide Schilirò, Giancarlo Cascarano, Luca Gualtieri, Anastasiya Valevich, Giulia Puccetti, Linda Carli, Chiara Stagnaro, Marta Mosca

**Affiliations:** 1 Rheumatology Unit, Department of Clinical and Experimental Medicine, University of Pisa, Pisa, Italy; 2 Department of Medical Biotechnologies, University of Siena, Siena, Italy

**Keywords:** Lupus Erythematosus, Systemic, Biological Therapy, Outcome Assessment, Health Care

## Abstract

**Objective:**

The objective is to evaluate perscriptions of belimumab (BEL), how these have changed over the years and their impact on clinical outcomes in patients with systemic lupus erythematosus (SLE).

**Methods:**

This is a retrospective analysis of prospectively collected data. We retrieved demographic and clinical data and concomitant therapies at BEL starting (baseline). Disease activity was assessed at baseline and after 6 and 12 months and organ damage at baseline and at the last visit.

**Results:**

From 422 patients followed in the Pisa SLE cohort, 102 patients received BEL and were included and 22 (21.6%) were immunosuppressant (IS)-naïve. Lupus Low Disease Activity State (LLDAS) with a glucocorticoid (GC) dosage ≤5 mg/day (LLDAS5) and remission were achieved by 47% and 38% of patients at 6 months, and by 75% and 66% at 12 months. Comparing IS-naïve patients with those who received BEL after at least one conventional IS, we did not find significant differences in baseline characteristics and in the achievement of LLDAS5 and remission. Despite at baseline we did not observe significant differences in mean GC daily dosage, IS-naïve patients were taking a significantly lower GC daily dose at 6 and 12 months. Interestingly, IS-naïve patients were more common in the most recent years.

**Conclusions:**

Our data confirm that BEL is effective in controlling disease activity, and in recent years BEL has been considered as an earlier treatment option before other IS. Early introduction of BEL can be at least as effective as a step-up approach and can help to reduce the GC dosage.

**WHAT IS ALREADY KNOWN ON THIS TOPIC**

- More than 10 years have passed since the approval of belimumab (BEL) for systemic lupus erythematosus (SLE), and data on real-life use of this drug are more and more emerging. However, little is known on the early use of biological drugs in extrarenal manifestations, especially in immunosuppressant (IS)-naïve patients.

**WHAT THIS STUDY ADDS**

- The approach to treatment with BEL seems to have changed over the years, as in the first years BEL was prescribed mainly to patients with a refractory disease treated with more than one IS drug, while in the most recent years it has been considered as an earlier treatment option before other ISs.
- In our cohort, we did not observe significant differences in response between patients treated or not treated with conventional IS drugs before BEL, but in IS-naïve patients we observed a significant glucocorticoid (GC) sparing effect at 6 and 12 months with respect to the other patients.

**HOW THIS STUDY MIGHT AFFECT RESEARCH, PRACTICE OR POLICY**

- These data suggest that BEL is effective in controlling disease activity and reducing the daily dose of GCs in SLE, and can be considered as an early treatment option before conventional IS.

## Introduction

 Systemic lupus erythematosus (SLE) is a systemic autoimmune disease with a wide range of clinical manifestations. The therapeutic armamentarium for the disease consists of glucocorticoids (GC), antimalarials, conventional immunosuppressants (IS) and more recently biological drugs.

Belimumab (BEL) is a recombinant, human, monoclonal antibody that binds to and antagonises soluble B-lymphocyte stimulator protein. In 2011, BEL was approved by the Food and Drug Administration for the treatment of adult patients with active, autoantibody-positive SLE who are receiving standard therapy, becoming the first biological agent to be approved for SLE. In Italy, BEL has been available since 2013.

More than 10 years have passed since the approval of BEL, and data accumulating show that BEL treatment is associated with improvements in disease activity, reduction in daily GC dose, reduction in flare incidence^1 2^ and prevention of damage progression.^3^

In the 2023 update of the EULAR recommendations, in patients with SLE with non-renal disease not responding to hydroxychloroquine (HCQ) or unable to reduce GCs below doses acceptable for chronic use (≤5 mg/day), the addition of conventional IS drugs or biological drugs (BEL and anifrolumab) is recommended and prior use of conventional IS drugs is not mandatory before initiating a biological agent.^4^ However, few data are available on the early use of biological drugs in extrarenal manifestations, especially in IS-naïve patients and the cost-effectiveness of this approach with respect to the traditional step-up has to be fully demonstrated. Starting from these promises, the objectives of this study were to evaluate the therapeutic trajectories of BEL, how these have changed over the years and what impact these changes have had on the achievement of the targets of remission and low disease activity as well as on other clinical outcomes.

## Methods

This is a retrospective analysis of prospectively collected data from adult patients with SLE satisfying the 2019 EULAR/American College of Rheumatology (ACR) classification criteria,^5^ regularly monitored at the Lupus Clinic of the University of Pisa.

All patients with SLE who started BEL between 2013 and 2023 in both intravenous and subcutaneous formulations for non-renal flare were eligible for inclusion.

For each patient, demographic and historic clinical data, active organ involvement and concomitant therapies at the initiation of BEL (baseline) were retrieved from clinical charts. Disease activity was assessed at baseline and after 6 and 12 months of therapy by means of the SLEDAI 2K score^6^; damage was assessed at baseline and at the last follow-up by means of the Systemic Lupus International Collaborating Clinics/ACR SLE Damage Index (SDI) score.^7^ Lupus Low Disease Activity State (LLDAS) defined on the basis of the Asian Pacific Lupus Consortium definition,^8^ LLDAS5^9^ and remission defined according to the 2021 DORIS (Definitions of Remission in SLE) criteria^10^ were evaluated as treatment targets. Briefly, LLDAS5 is a variation of the classic definition of LLDAS by Franklyn *et al*^8^ which includes GC dosage ≤5 instead of 7.5 mg/day, previously proposed by our group.^9^

We considered IS-naive patients who had received only HCQ and GCs prior to BEL, and the term ‘IS’ used through the manuscript includes conventional IS drugs, immunomodulatory drugs as methotrexate (not including HCQ) and biological drugs excluding BEL.

### Statistical analysis

Normality of continuous variables was assessed through the Shapiro-Wilk test. Continuous variables were described as median and 25–75 IQR or as mean and SD, as appropriate.

Categorical variables are reported as proportions. Cross-tabulated data were analysed using χ^2^ test and Student’s t-test and comparisons between groups were performed using the Mann-Whitney test. P values less than 0.05 were considered as statistically significant.

The probability of retention of BEL treatment was assessed using Kaplan-Meier survival analysis, and differences according to prescription group were evaluated using the log-rank test.

Informed consent was obtained by all patients and was registered on medical records.

## Results

From a total of 422 patients regularly followed in the Pisa SLE cohort, 102 patients (24%) received BEL for non-renal flares from 2013 to 2023 and they have been included in this analysis. Of our cohort, 30 patients (29%) have started intravenous BEL and currently 5 are continuing therapy in that formulation, while the others are taking subcutaneous BEL.

They were predominantly female (n=96, 94%) and Caucasian (n=96, 94%); mean age at diagnosis was 28.7±11.4 years, and mean age and mean disease duration at baseline were 38.6±11.6 and 10.1±8.6 years, respectively. The median duration of follow-up after the introduction of BEL was 28.5 months (IQR 12–42.7) and demographic and clinical data, including active organ involvement at baseline, are summarised in table 1.

**Table 1:** Characteristics of the cohort (n=102) and comparison between immunosuppressants-naïve (group 1) and the other patients (group 2) at start of belimumab therapy

	Whole cohort n=102	Group 1n=22	Group 2n=80	P value
Age at diagnosis, years*	28.7±11.3	30.7±11.2	28.1±11.4	0.35
Female (%)	96 (94)	19 (86)	77 (96)	0.08
Overlap syndrome (%)	29 (28)	5 (23)	24 (30)	0.50
2019 EULAR/ACR criteria count*	23.1±9.9	21.9±5.9	23.5±10.7	0.50
Concomitant hydroxychloroquine (%)	81 (79)	19 (87)	62 (88)	0.36
Age at baseline, years*	38.6±11.5	39.3±11.5	38.4±11.6	0.73
Disease duration at baseline, years*	10.1±8.6	8.63±9.1	10.5±8.4	0.34
SLEDAI at baseline*	7.2±3.4	8.0±4.7	7.0±3.0	0.25
Anti-dsDNA positivity at baseline (%)	78 (77)	16 (73)	62 (78)	0.64
Glucocorticoids mg/day at baseline*	7.7±7.3	5.8±4.2	8.2±7.9	0.29
Constitutional involvement (%)†	31 (30)	6 (27)	25 (31)	0.43
Joint involvement (%)†	72 (71)	17 (69)	55 (69)	0.43
Skin involvement (%)†	48 (47)	12 (55)	36 (43)	0.60
Renal involvement (%)†	14 (14)	1 (8)	13 (16)	0.15
Pulmonary involvement (%)†	3 (3)	0 (0)	3 (4)	0.35
Haematological involvement (%)†	27 (27)	7 (32)	15 (19)	0.52
Serositis (%)†	7 (7)	1 (8)	6 (8)	0.62
Neuropsychiatric involvement (%)†	3 (3)	0 (0)	3 (4)	0.35

* Mean±standard deviationSD; * active at the time of belimumab start..

† Active at the time of belimumab start.

ACRAmerican College of RheumatologyEULAREuropean Alliance of Associations for Rheumatology

**Figure 1:**
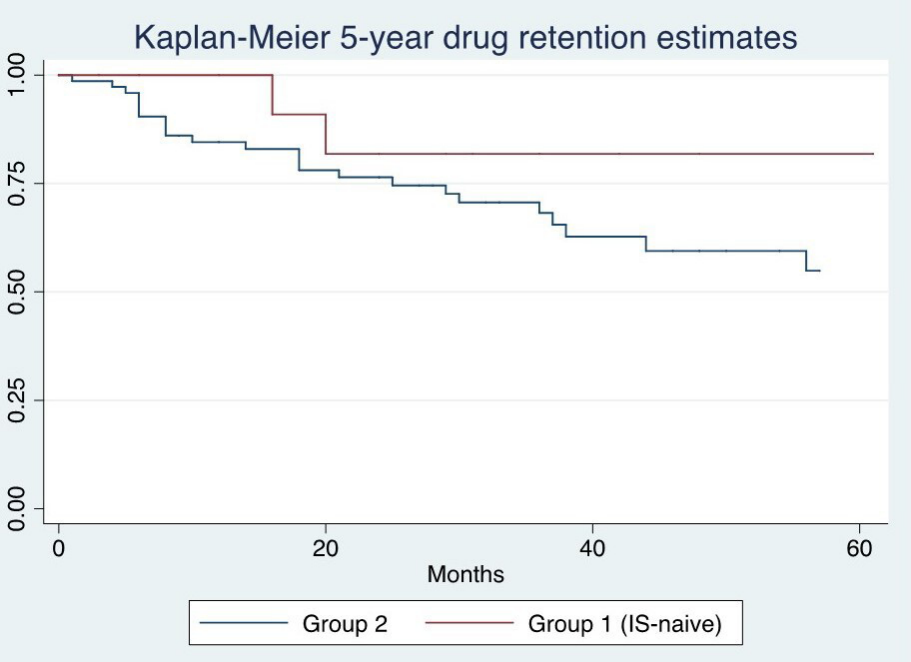
Belimumab retention estimates.

During the follow-up, 29 (28%) patients discontinued BEL. Reasons for discontinuation were inefficacy (18%, n=18), adverse events (8%, n=9) and sustained remission status (2%, n=2). Adverse events included two cases of allergic reactions, two severe neutropenia, two cases of headache not responding to anti-inflammatories, one case of depression with suicidal ideation, one case of thyroid cancer and one case of recurrent infections since BEL starting. Treatment retention estimates are reported in figure 1, and no differences were found between groups 1 and 2 (p=0.33).

### Treatment prescription

**Figure 2:**
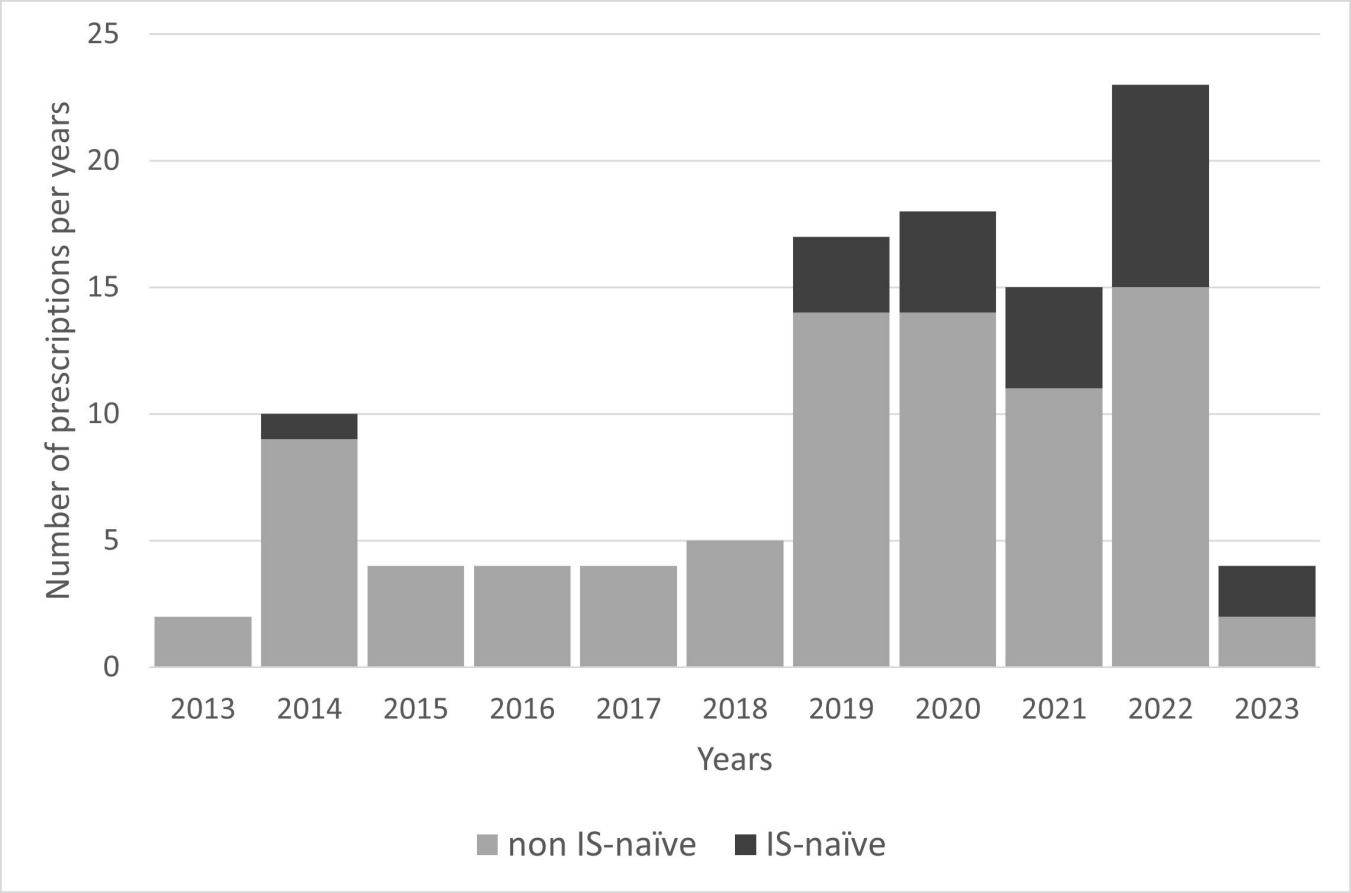
Incidence of patients immunosuppressants (IS)-naïve according to years of prescription since belimumab availability.

In the first 5 years since BEL availability, the drug was mainly prescribed in patients with a refractory disease previously treated with more than one IS, and only one case of IS-naïve patient was observed in 2014 (figure 2). After 2018, the number of IS-naïve patients increased as well as the number of patients who tried a lower number of conventional IS with respect to the previous years. As a matter of fact, in patients not IS-naïve who started BEL from 2013 to 2018, mean number of conventional IS received before BEL was 2.14±1.5 versus 1.2±1.1 in patients who started BEL from 2019 to 2013 (p<0.01).

Overall, in our cohort, 22 patients (22%) were IS-naïve, while the other 80 (78%) received at least 1 conventional IS before BEL (mean 1.8±1.2, min 1–max 6).

Fifty-seven patients (56%) received BEL as monotherapy (other than HCQ), while 45 (44%) received a concomitant conventional IS. 17 patients who received BEL as monotherapy were also IS-naïve. IS therapies received before BEL in our cohort are summarised in table 2.

**Table 2:** Immunosuppressive/DMARD (disease modifying anti-rheumatic drugs) therapies received before belimumab in patients not IS-naïve (n=80)

Drug	Patients (%)
Azathioprine (%)	29 (36)
Mycophenolate (%)	23 (29)
Methotrexate (%)	39 (49)
Cyclophosphamide (%)	15 (19)
Cyclosporine (%)	20 (25)
Other (%)	4 (5)

ISimmunosuppressants

Concomitant therapies included GCs in 78% (n=79; mean daily dose prednisone equivalent 7.8±7.3) and HCQ in 79% (n=81). Regarding the 79 patients who received GC, in 33 (32%) the GC dosage was increased to treat the flare (mean daily dose 15.3±11.9, median 10 IQR 10–15) while the remaining 46 (45%) maintained the same GC dosage. The other 23 patients (23%) did not receive systemic GC to treat the disease flare.

Comparing IS-naïve patients (group 1) and patients who received BEL after at least one conventional IS (group 2), we did not find significant differences with regard to age, disease duration, type of organ involvement, SLEDAI-2K and anti-dsDNA positivity at treatment start (table 1).

No differences were also found in the concomitant GC dosage at BEL start between the two groups; 15 (68.2%) patients were under GC treatment at baseline with a mean dose of 5.8±4.2 mg/day prednisone equivalent in group 1 and 64 (80%) in group 2 with a mean daily dosage of 8.2±7.9 (p=0.29); similarly, the percentages of patients with a daily GC dose of ≤5 mg prednisone equivalent at baseline resulted 70% in group 1 and 62.4% in group 2.

### 6 and 12 months outcomes

LLDAS5 and remission were achieved by 47% and 38% of patients at 6 months, and by 74% and 66% at 12 months, respectively. At 6 months, only 4 patients were in LLDAS but not LLDAS5 because of a daily GC dosage of 7.5 mg, and at 12 months of follow-up all patients in LLDAS satisfied also LLDAS5 definition. Interestingly, no differences were found in the achievement of LLDAS5 and remission at 6 months (61% vs 42% p=0.15 and 50% vs 35% p=0.24) and at 12 months (60% vs 80% p=0.13 and 60% vs 68% p=0.56) between patients IS-naïve and not IS-naïve respectively.

Moreover, despite at baseline we did not observe significant differences in mean GC daily dosage between the two groups, at 6 and 12 months the subgroup of patients IS-naïve were taking a significantly lower GC daily dose (3.0 vs 5.5 p=0.02, 2.3 vs 4.3 p<0.01).

At 6 and 12 months, the percentages of patients with a current daily GC dose of ≤5 mg prednisone equivalent resulted 89% and 100% in group 1, and 77% and 93% in group 2 (p=0.22).

Comparison between the two groups is detailed in table 3. (Table 3)

**Table 3:** 6 and 12 months outcomes in patients who reached these timepoints

	Whole cohort n=102	Group 1n=22	Group 2n=80	P value
LLDAS at 6 months (%)	38/73 (52)	11/18 (61)	27/55 (49)	0.20
LLDAS5 at 6 months (%)	34/73 (47)	11/18 (61)	23/55 (42)	0.15
Remission at 6 months (%)	28/73 (38)	9/18 (50)	19/55 (35)	0.24
Glucocorticoids mg/day at 6 months^*^	4.8±3.0	3.0±2.6	5.5±3.0	**0.02**
LLDAS at 12 months (%)	44/59 (75)	9/15 (60)	35/44 (80)	0.13
LLDAS5 at 12 months (%)	44/59 (75)	9/15 (60)	35/44 (80)	0.13
Remission at 12 months (%)	39/59 (66)	9/15 (60)	30/44 (68)	0.56
Glucocorticoids mg/day at 12 months^*^	3.8±2.4	2.3±1.9	4.3±2.4	**<0.01**
SDI ≥1 in patients with at least 12 months of follow-up	10/59 (17)	1/15 (7)	9/44 (21)	0.42
Flare over a maximum of 12 months follow-up (%)	14/72 (19)	1/15 (7)	13/57 (23)	0.27

Statistically significant values are in bold.

* Mean±standard deviation

LLDASLupus Low Disease Activity StateSDISLE Damage Index

In table 3patients with a follow-up of at least 12 months after the introduction of BEL (n=59), we observed an increase in SDI ≥1 in 10 patients (17%). Of them, only one patient was in IS-naïve group while the other patients were in group 2 (7% vs 21%, p=0.42).

## Discussion

In the present study, we have described treatment trajectories of BEL in a cohort of patients with SLE, their changes over time and the impact of these changes on clinical outcomes and targets.

Since BEL availability in Italy, we noticed a significant change in the prescription approach over the years, as in the first years BEL was prescribed mainly to patients with a refractory disease treated with more than one IS drug, while over the last years the percentage of IS-naïve patients who received BEL increased significantly.

We did not find significant differences in terms of baseline clinical manifestations between IS-naïve patients and the other group, meaning that in the IS-naïve cohort the decision to prescribe BEL as second-line treatment after HCQ and GC failure was not based on a specific patient’s clinical profile. The main difference, however, resided in the fact that in the early phase of the new drug availability, refractory patients, who failed several prior conventional therapies, were the first candidates to receive BEL, as supported by the number of previous IS therapies.

This is not surprising considering that it is well known that the time to adoption of new drugs is influenced by a wide variety of factors, which include both physicians and patients variables^11^ and adoption can go to few months^12^ to 2 or more years^13^ from the availability of the drugs. Moreover, these results can be explained by the development of recommendations for SLE management; as in the 2019 update^14^ BEL was included among the treatment options confined in the moderate-refractory manifestations (possibly after conventional IS) and only in the most recent update its indication has been extended to mild–moderate–severe forms even without previous conventional IS.

As far as the treatment outcome is concerned, in our real-world study, we observed few cases of discontinuations related to inefficacy or adverse events, and we observed a good efficacy of BEL treatment with achievement of LLDAS and/or remission by a good percentage of patients at 6 and 12 months from baseline. Our results also showed that the time to reach the targets in IS-naïve patients seems to be shorter than in patients included in group 2, especially for remission. Although this observation should be confirmed in larger studies, this is an important point as the time to response in crucial in the treat-to-target approach. A new observation of our cohort is the attainment of LLDAS5, a definition of low disease activity with 5 mg of prednisone instead of 7.5 mg/day which we have proposed and evaluated in 2018.^9^ In our cohort, LLDAS5 was attained by about 62% of patients^9^ and today it appears in line with the 2023 update of European Alliance of Associations for Rheumatology Recommendations, in which a prednisone dose ≤5 mg/day is recommended as a maintenance dose.

Our findings are in line with earlier cohorts demonstrating significant improvement in disease activity indices during BEL treatment and a significant attainment of LLDAS and remission states.

Gatto *et al*^15^ in a large Italian cohort of patients treated with BEL reported 55.6% and 49.2% of attainment of LLDAS and remission, respectively, at 6 months of treatment and 71.7% and 41.1% at 12 months.

In 2020, Sbeih *et al*^16^ also reported a probability of reaching LLDAS or remission at month 12 of 58.1% and 37.1%, respectively; however, in this cohort, only patients refractory to multiple IS drugs (minimum number of previous IS was 2 and maximum 9) were included.

More recently, in a similar real-life setting of a multicentric cohort, Nikoloudaki *et al*^17^ described lower percentages of LLDAS and remission attainment both at 6 (36.2% and 13.2% respectively) and at 12 months (36.7% and 11.6% respectively). Of note, the vast majority of the patients enrolled in this cohort (95.7%) had been previously treated with IS or biological agents including cyclophosphamide and rituximab while IS-naïve patients were not included.

Of novelty with respect to previous cohorts, our study included a significant percentage of patients IS-naïve and no significant differences were observed in response between patients treated or not treated with conventional IS drugs before BEL; these data suggest that BEL as the first choice IS therapy is able to control disease activity without the need to add traditional IS drugs.

An additional interesting finding in our study refers to the analysis of GC treatment trajectories in the two groups; indeed, in cases of IS-naïve patients, we observed a significant GC-sparing effect at 6 and 12 months with respect to the other patients.

The steroid-sparing effect of BEL is well established in the literature; in the previous cited studies by Nikoloudaki *et al*,^17^ a high percentage of patients (70%) was in a GC dosage <5 mg/day at 6 months after starting BEL, and Gatto *et al*^15^ reported a mean total daily intake of prednisone of 10.6±8.6 mg at baseline which decreased to 5.28±4.67 at 12 months.

Similarly to our study, Zhao *et al*^18^ recently showed that newly diagnosed patients with SLE taking BEL had the highest rate of achievement of SRI-4 (SLE esponder Index) Responder Index) with respect to relapsing and refractory patients; moreover, the newly diagnosed subgroup demonstrated a significant GC sparing.

The novelty of our finding with respect to previous literature, however, resides in the fact that this effect is clear among patients who have been treated with BEL monotherapy and naïve to IS drugs.

Finally, we observed a low damage progression in IS-naïve patients, since only 1 patient (7%) of this subgroup had an increase in SDI score during a follow-up of at least 12 months, while in the other group we observed 9 cases (21%) of damage progression. Although this difference is not statistically significant, probably due to the low number of observations included, this suggests a BEL disease-modifying effect in these patients.

These data suggest that the early introduction of biological drugs in SLE could be an effective option to help to achieve the targets proposed in the treat-to-target recommendations for SLE,^19^ such as control of disease activity, prevention of damage and the use of low GC doses.

We have to acknowledge that our study has some limitations. Due to the study design and the absence of a comparison group with standard of care, our data cannot demonstrate that early introduction of BEL is superior to standard of care (including non-biological drugs) in steroid sparing. In addition, this is a retrospective study; however, our data derived from a real-life practice of a SLE referral with a careful prospective collection of clinical data.

On the other hand, one of the strengths of this study is that it reflects the real-world setting, and this makes the results of the study generalisable to day-to-day practice.

To our knowledge, this is the first study analysing changes of treatment trajectories of BEL over time in a cohort of predominantly European ancestry patients. The study also shows, in a real-world setting, that an early introduction of BEL in IS-naïve patients appears to have the same positive effects on outcomes as the add-on approach, where BEL is added on top of a traditional IS drugs, with a significant GC-sparing effect.

In conclusion, our study confirms that BEL is effective in controlling disease activity in SLE and reducing the daily dose of GCs, and can be considered as an early treatment option also before conventional IS.

## Data Availability

Data are available upon reasonable request.
